# Bibliometric indicators of the *Croatian Medical Journal* – can small non-profit journal compete in the bibliometrics arena?

**DOI:** 10.3325/cmj.2022.63.501

**Published:** 2022-12

**Authors:** Nataša Kovačić, Jelka Petrak

**Affiliations:** 1 *Croatian Medical Journal*, University of Zagreb, School of Medicine, Zagreb, Croatia; 2 Department of Anatomy and Clinical Anatomy, University of Zagreb, School of Medicine, Zagreb, Croatia

Scientific journals from small scientific communities face many obstacles, ranging from an insufficient inflow of qualitative manuscripts to a modest choice of skilled reviewers and limited international visibility (1). As bibliometrics is currently the central plank in the assessment of the scientific output (2), authors are inclined to publish in well-established core journals.

Since its establishment, editors of the *Croatian Medical Journal* (*CMJ*) have invested enormous effort to attract internationally recognized authors, increase the quality of domestic submissions, and improve the review process by recruiting well-educated local reviewers, continuously educating both potential authors and reviewers. In addition, an important step in achieving international visibility was the inclusion in the international bibliographic databases. The *CMJ* has been covered by the Scopus database since 1992, with 2580 articles indexed until 2021 (1992 and 1993 were represented selectively, with only 9 and 10 articles, respectively). Since 1998, the *CMJ* has been indexed in Medline/PubMed, and since 1999 in Current Contents/Clinical Medicine and Science Citation Index Expanded, with more than 2300 articles available in these databases. Publishing in open access also increases international visibility of a journal, so the *CMJ* uses the Diamond Open Access model, with no author processing fees or fees to access the published articles. Besides the *CMJ*'s web site, the journal has since 2006 been freely available on PubMed Central (full-participation base) and on the Hrčak portal, a local platform for Croatian scientific and technical journals.

**Figure 1:**
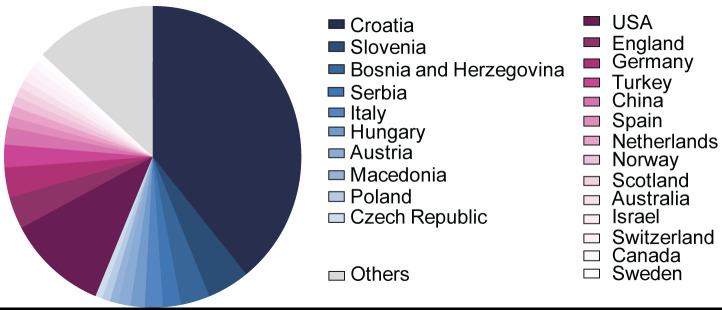
Distribution of the *Croatian Medical Journal* articles indexed in the Science Citation Index Expanded according to their geographical origin. Data were retrieved by searching the Web of Science Core Collection (on 11/10/2022) for the publication title “Croatian Medical Journal.” “Countries/Regions” represented with 20 or more items (24 countries) are specified, while countries with fewer than 20 items (total 368 articles from 71 countries) are represented as “others,” among 2731 items.

One of the goals of the *CMJ* has been to represent a gateway for Croatian medical researchers to the global community. The articles from Croatian authors account for approximately 40% of all the journal’s entries in Science Citation Index Expanded and 47% in Scopus. In addition, the *CMJ* has also attracted articles from neighboring and regional countries, which together with the articles by Croatian authors, account for more than a half (56%) of the journal contributions (Figure 1).

**Figure 2:**
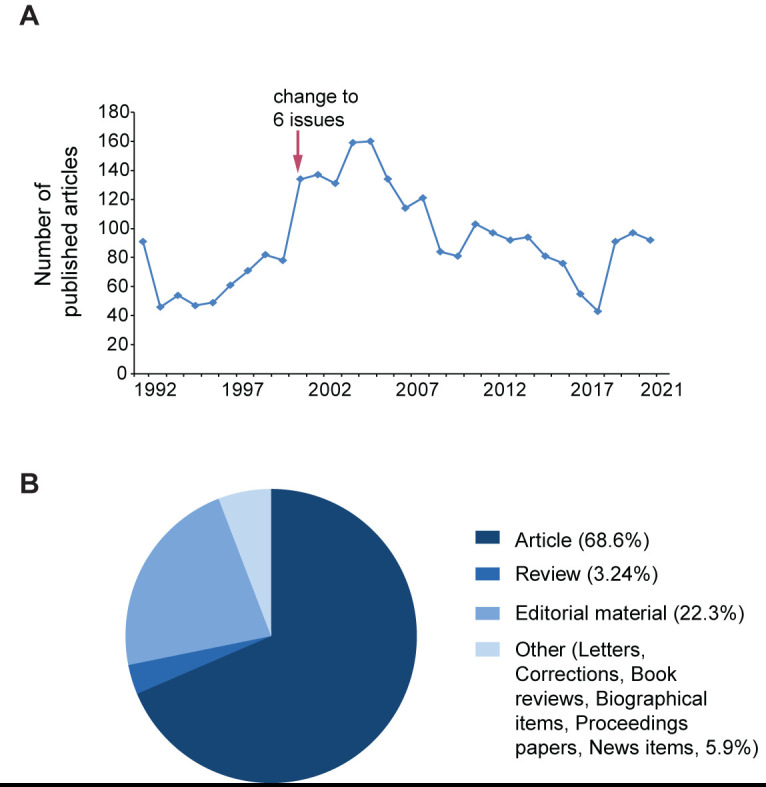
The number of articles published in the *Croatian Medical Journal* over 30 years. (**A**) total number of published articles, (**B**) distribution of different types of articles among all articles indexed in the Science Citation Index Expanded. Data were retrieved by searching the Web of Science Core Collection (on 11/10/2022) by the publication title “*Croatian Medical Journal*.” Distribution of articles is shown according to WoS “Document types.” The number of articles published in years before the inclusion in Science Citation Index Expanded (**A**) was determined manually (5).

The annual number of articles published in the *CMJ* has varied over time, reflecting changes in editorial policies (Figure 2A). It peaked between 2001 (when the journal switched from four to six issues per year) and 2008. After that, the number has remained under 100. As journals’ influence is often assessed through the number of citations and impact factor (IF), many journal editors considered adjusting their policies to increase these metrics. Some examples of these adjustments are increasing the proportion of review articles, which are expected to be more cited, or increasing the proportion of non-citable items, such as letters and editorials, which receive citations but are not included among citable items during IF calculation (3). The *CMJ*’s editorial policy has been mainly targeted at the improvement of writing skills of local authors, and encouraging authors to submit high-quality reports. In line with the journal’s scope (4), with preference toward original research results, the majority of published items are original articles (Figure 2B).

**Figure 3:**
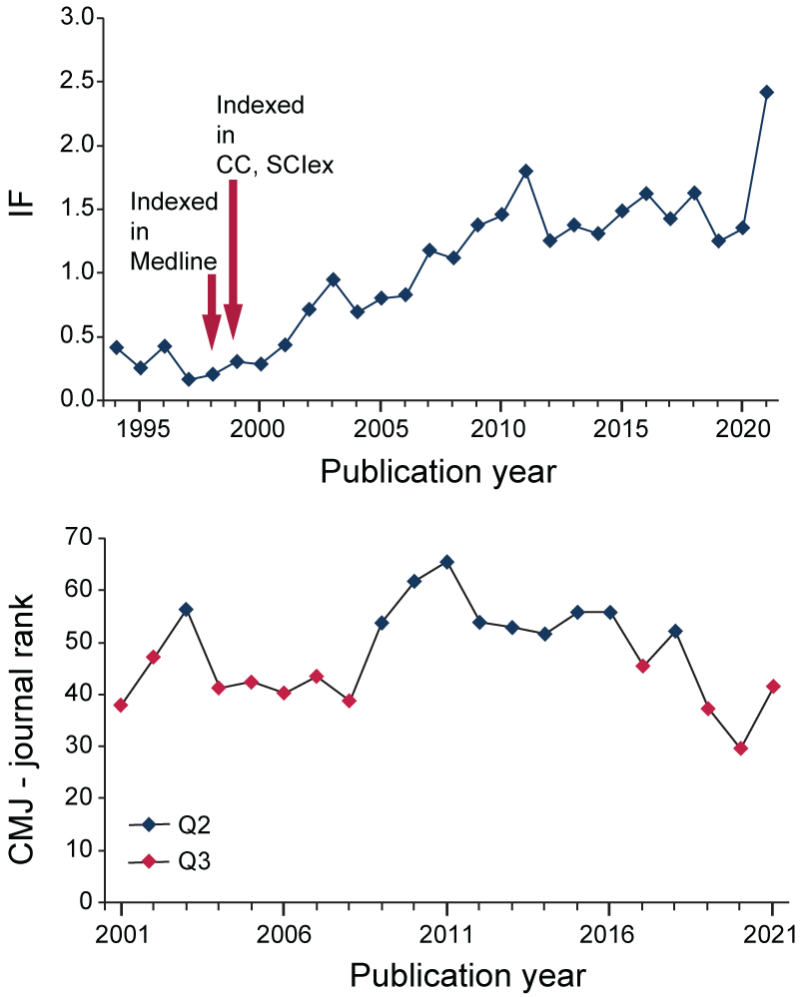
Two-year impact factor (IF) of the *Croatian Medical Journal* (**A**) and journal percentile rank in the Science Citation Index Expanded category Medicine, General & Internal, available since 2001 (**B**). Data were retrieved from Journal Citation reports (on 12/10/2022). The IF before 2001 (**A**) was calculated manually (5).

Despite many known limitations and fierce criticism (6), the IF of a journal and its quartile (Q) rank in the subject category remain the most important criteria when it comes to assessing the journal quality. Since the *CMJ*’s founding, the journal’s IF has increased substantially, peaking in 2021 (Figure 3A). However, the journal remains in the third quartile (119 out of 172 journals) in its category – Medicine, General & Internal. The best rank was achieved between 2009 and 2011, and the journal remained in the second quartile until 2016 (Figure 3B). The field of biomedicine, Medicine, General & Internal is one of the most competitive categories; besides the highest ranking journal with an IF of 202.7, more than 20 journals have IFs greater than 10. However, even in Q3, the *CMJ*'s ranks higher than many well-established medical journals with a long publishing history. Therefore, assessing journal quality based on its rank relies on an arguable assumption that a few citations make a big difference (7). In addition, the total number of journals in this category increased from 112 in 2001 to 172 in 2021, so the decrease in the *CMJ*'s rank may be attributed to a rapid increase in IF of more recently included open-access journals over the last decade.

**Figure 4:**
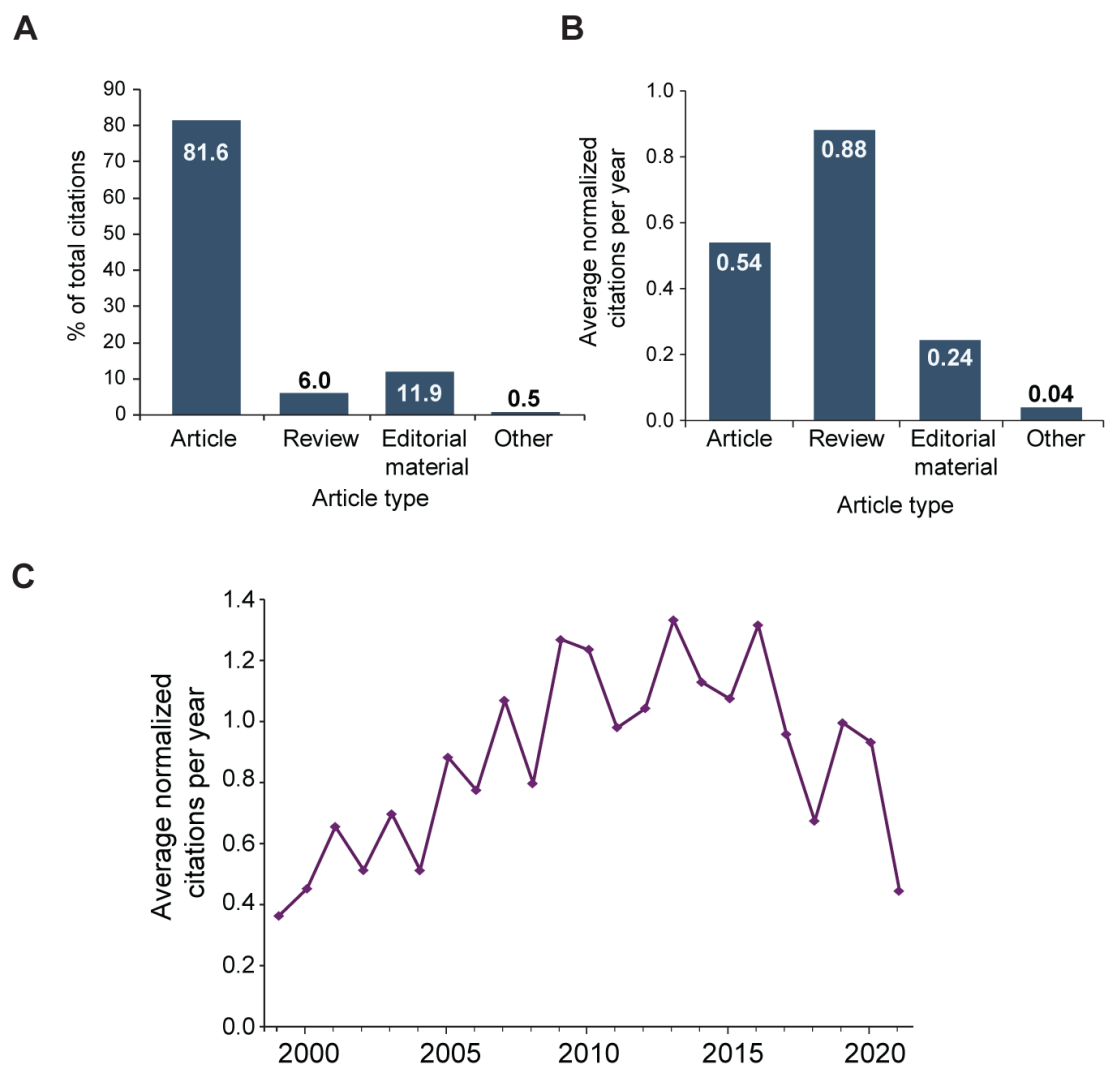
(**A**) Distribution of total citations, (**B**) normalized citations among article types, and (**C**) normalized citations over time. Data were retrieved by searching the Web of Science Core Collection (on 13/10/2022) for the publication title “Croatian Medical Journal,” subsequent filtering according to “Document types” or “Publication years,” and retrieving the citation report for each filter. The number of articles and average number of citation per year were used for calculation and graphical presentation. Normalized citations were calculated (**B**) by dividing the average number of citations per year acquired by each type of article with the total number of articles of the same type, or (**C**) by dividing the average number of citations per year with the total number of articles published in the same year.

Since its establishment, the *CMJ* has earned 25 891 citations (92.6% journal-independent citations). The majority of these citations were received by items classified as articles (81.6%) and editorial material (11.9%), in line with their frequencies in the total number of articles (Figure 4A). However, when the average number of citations per year is normalized according to the article type, reviews attracted more citations, followed by original articles, while each editorial material item received on average one citation in four years (Figure 4B). The average normalized number of citations per year for all articles is 0.45, so each article published in the *CMJ* was cited approximately once in two years. However, articles published between 2007 and 2017 were cited approximately once a year, pointing to an overall increase in the citation rate (Figure 4C).

The *CMJ* articles are characterized by a long cited half-life, an indicator representing the median age of articles cited in a particular year (8). According to Journal Citation Reports (JCR), the cited half-life of the *CMJ* in the recent decade fluctuated between 6.1 and 10. In the current, 2021, edition of JCR the *CMJ* is among the 30 journals with the longest half-life in the category Medicine, General & Internal. On the other hand, the immediacy index, an indicator representing the number of citations that articles attracted in the publication year, was low (between 0.1 and 0.6). According to the immediacy index, the *CMJ* in the 2021 edition of JCR ranked the same as according to the impact factor (119 out of 172 journals in category Medicine, General & Internal). Clearly, the articles published in the *CMJ* need more time to receive citations, but also remain cited for a long time, so the normalized citation number of recent issues is expected to increase. This is also evident when ranking the articles according to their total, vs average, citation numbers. Some of the more recent articles with highest average normalized citation number per year, although at the moment have fewer total citations, in future might become most cited according to both criteria (Table 1).

**Table 1:** Articles with the highest total number of citations and normalized average number of citations per year

R1*	R2*	Authors	Title	Year	Total citations^†^	Average citations/y^‡^
1	3	Godfroid J, Nielsen K, Saegerman C.	Diagnosis of brucellosis in livestock and wildlife	2010	154	11.85
2	2	Jackson S, Mathews KH, Pulanic D, et al.	Risk factors for severe acute lower respiratory infections in children - a systematic review and meta-analysis	2013	137	13.7
3	16	Alonso A, Andelinovic S, Martin P, et al.	DNA typing from skeletal remains: Evaluation of multiplex and megaplex STR systems on DNA isolated from bone and teeth samples	2001	137	6.23
4	45	Rudan I, Gibson JL, Ameratunga S, et al.	Setting priorities in global child health research investments: guidelines for implementation of CHNRI method	2008	130	8.67
5	4	Budowle B, Eisenberg AJ, van Daal A.	Validity of low copy number typing and applications to forensic science	2009	128	9.14
6	25	Gill P.	Application of low copy number DNA profiling	2001	127	5.77
7	19	Bossuyt PM, Reitsma JB, Bruns D, et al.	The STARD statement for reporting studies of diagnostic accuracy: Explanation and elaboration	2003	123	6.15
8	9	Gwida M, Al Dahouk S, Melzer F, Roesler U, Neubauer H, Tomaso H.	Brucellosis - regionally emerging zoonotic disease?	2010	110	8.46
9	21	Bostanci M, Ozdel O, Oguzhanoglu NK et al.	Depressive symptomatology among university students in Denizli, Turkey: Prevalence and sociodemographic correlates	2005	106	5.89
10	24	Hranjec T, Kovac A, Kos J, et al.	Endemic nephropathy: the case for chronic poisoning by Aristolochia	2005	104	5.78
11	17	Davoren J, Vanek D, Konjhodzic R, et al.	Highly effective DNA extraction method for nuclear short tandem repeat testing of skeletal remains from mass graves	2007	99	6.19
12	37	Holland MM, Cave CA, Holland CA, Bille TW.	Development of a quality, high throughput DNA analysis procedure for skeletal samples to assist with the identification of victims from the world trade center attacks	2003	99	4.95
13	20	Milos A, Selmanovic A, Smajlovic L, et al.	Success rates of nuclear short tandem repeat typing from different skeletal elements	2007	98	6.13
14	42	King DR, Kanavos P.	Encouraging the use of generic medicines: Implications for transition economies	2002	97	4.62
15	43	Ozcankaya R, Delibas N.	Malondialdehyde, superoxide dismutase, melatonin, iron, copper, and zinc blood concentrations in patients with Alzheimer disease: cross-sectional study	2002	97	4.62
16	35	Alonso A, Martin P, Albarran C et al.	Challenges of DNA profiling in mass disaster investigations	2005	90	5
17	13	Yu WL, Nielsen K.	Review of detection of Brucella spp by polymerase chain reaction	2010	88	6.77
18	29	Kane GC, Gotto JL, Mangione S, West S, Hojat M.	Jefferson Scale of Patient's Perceptions of Physician Empathy: Preliminary psychometric data	2007	88	5.5
19	7	Adeloye D, Chan KY, Rudan I, Campbell H.	An estimate of asthma prevalence in Africa: a systematic analysis	2013	87	8.7
20	80	Marušić A, Marušić M.	Small scientific journals from small countries: Breaking from a vicious circle of inadequacy	1999	84	3.5
27	18	Budimir D, Polasek O, Marušić A, et al.	Ethical aspects of human biobanks: a systematic review	2011	74	6.17
41	6	Venkat P, Chopp M, Chen J.	New insights into coupling and uncoupling of cerebral blood flow and metabolism in the brain	2016	62	8.86
69	1	Dogas Z, Kalcina LL, Dodig IP, et al.	The effect of COVID-19 lockdown on lifestyle and mood in Croatian general population: a cross-sectional study	2020	51	17
77	12	Pelcic G, Karacic S, Mikirtichan GL, et al.	Religious exception for vaccination or religious excuses for avoiding vaccination	2016	48	6.86
124	14	Svalastog AL, Donev D, Kristoffersen NJ, Gajovic S.	Concepts and definitions of health and health-related values in the knowledge landscapes of the digital society	2017	38	6.33
171	10	Hudetz D, Borić I, Rod E, et al.	Early results of intra-articular micro-fragmented lipoaspirate treatment in patients with late stages knee osteoarthritis: a prospective study	2019	32	8
201	11	Adani S, Cepanec M.	Sex differences in early communication development: behavioral and neurobiological indicators of more vulnerable communication system development in boys	2019	30	7.5
243	5	Civljak R, Markotic A, Kuzman I.	The third coronavirus epidemic in the third millennium: what's next?	2020	27	9
278	15	Milo R.	Therapies for multiple sclerosis targeting B cells	2019	25	6.25

*R1, rank of the article according to total number of acquired citations; R2, rank of the article according to the number of normalized citations per year

†Numbers of total citations, and ‡average citations per year were retrieved by searching the Web of Science Core Collection (all editions, 13/10/2022) according to the publication title “Croatian Medical Journal.”

**Figure 5:**
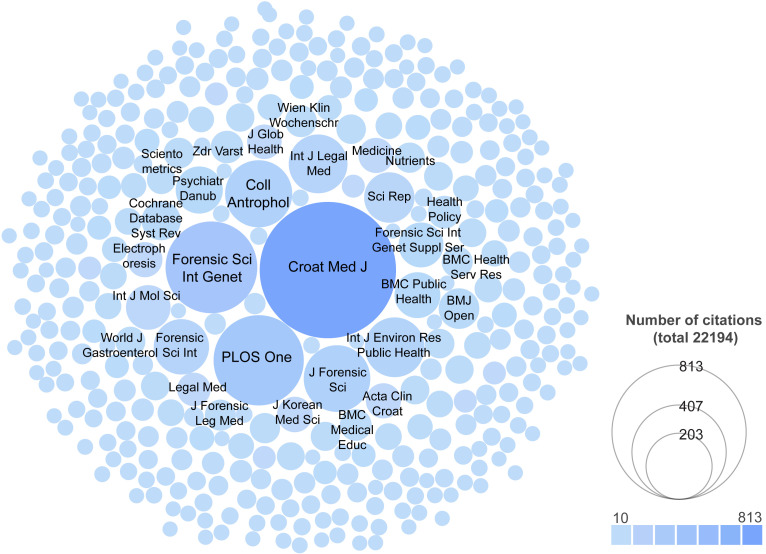
The citation network of the *Croatian Medical Journal* (*CMJ*) showing 369 journals that cited articles from the *CMJ*≥10 times (9121 citing articles or 40.45% of 22 549 items). The 30 most represented journals are labeled. The intensities and sizes of the circles correspond to the number of cited articles. Graphical representation is produced by SCImago Graphica (9).

Besides the number of citations and IF, a journal's influence in overall scientific community is reflected in Eigenfactor score and Article Influence Score (8). These are weighted indicators that, together with the number of citations, take into account also the source of citations. Therefore, the weight assigned to citations from high-impact journals is greater than the weight of citations from the low-impact journals. According to Eigenfactor score, the *CMJ* ranked 137th, and according to article influence score, 120th out of 172 journals in the category Medicine, General & Internal. This is not surprising since the *CMJ* is predominantly cited in journals from the subject categories with lower citation rates and IFs, such as forensics, or in mid-ranked general medical or research journals (Figure 5).

Since the establishment of the *CMJ*, editors have monitored the citation rates and IF (5,10). In the first fifteen years, marked by development and expansion of the journal, citation rates, IF, and journal rank in its subject category were continuously increasing. Over the recent fifteen years, indicators have varied depending on fluctuations in editorial policies, but also depending on external factors. Recent decades have seen an explosion of open-access journals, some quickly achieving positions in Q1 and high IFs. Additional imbalance was created by a recent surge in COVID-related research (11). Thus, there is a trend of overall decrease in rank even for reputable scientific journals with high quality requirements and rigorous peer review; this affects small, peripheral journals even more. Non-profit journals often do not possess the required infrastructure and personnel to compete with the production rates and outreach strategies of professional publishers charging for article processing.

Over the last 30 years, the *CMJ* has continuously focused on a rigorous peer-review process, and on attracting high-quality articles. This policy a decade ago led to satisfactory metric indicators, and now the question is whether it is possible or necessary to increase them further while staying aligned with the main values of the journal. Metric indicators are still among the main criteria for evaluating the scientific contribution of authors, projects, and institutions, and authors prefer publishing in journal “brands,” so in this game small journals do not stand a chance. However, increased awareness about shortcomings of purely bibliometric assessment, and recent requirements for reforming the evaluation of research output (12) will hopefully improve the position of the *CMJ* and other journals that insist on best editorial practices and rely on “passion for publishing and publishing with passion” (13).
